# Structure and stability of the *Human respiratory syncytial virus* M_2–1_ RNA-binding core domain reveals a compact and cooperative folding unit

**DOI:** 10.1107/S2053230X17017381

**Published:** 2017-12-15

**Authors:** Ivana G. Molina, Inokentijs Josts, Yasser Almeida Hernandez, Sebastian Esperante, Mariano Salgueiro, Maria M. Garcia Alai, Gonzalo de Prat-Gay, Henning Tidow

**Affiliations:** a Protein Structure–Function and Engineering Laboratory, Fundación Instituto Leloir and IIBBA–CONICET, Avenida Patricias Argentinas 435, C1405BWE Buenos Aires, Argentina; b The Hamburg Centre for Ultrafast Imaging and Department of Chemistry, Institute for Biochemistry and Molecular Biology, University of Hamburg, Martin-Luther-King-Platz 6, 20146 Hamburg, Germany; c European Molecular Biology Laboratory (EMBL), Hamburg Unit, Notkestrasse 85, 22607 Hamburg, Germany

**Keywords:** viral proteins, crystal structure, small-angle X-ray scattering, protein folding, *Human syncytial respiratory virus*

## Abstract

*Human syncytial respiratory virus* is a nonsegmented negative-strand RNA virus with serious implications for respiratory disease in infants, and has recently been reclassified into a new family, *Pneumoviridae*. One of the main reasons for this classification is the unique presence of a transcriptional antiterminator, called M_2–1_. The puzzling mechanism of action of M_2–1_, which is a rarity among antiterminators in viruses and is part of the RNA polymerase complex, relies on dissecting the structure and function of this multidomain tetramer. The RNA-binding activity is located in a monomeric globular ‘core’ domain, a high-resolution crystal structure of which is now presented. The structure reveals a compact domain which is superimposable on the full-length M_2–1_ tetramer, with additional electron density for the C-terminal tail that was not observed in the previous models. Moreover, its folding stability was determined through chemical denaturation, which shows that the secondary and tertiary structure unfold concomitantly, which is indicative of a two-state equilibrium. These results constitute a further step in the understanding of this unique RNA-binding domain, for which there is no sequence or structural counterpart outside this virus family, in addition to its implications in transcription regulation and its likeliness as an antiviral target.

## Abstract 2

A high-resolution crystal structure of the M_2–1_ RNA-binding domain of *Human syncytial respiratory virus* was determined. A combination of crystallography and SAXS indicated a role in its C-terminal extension.

## 1. Introduction   

*Human respiratory syncytial virus* (HRSV) is a nonsegmented negative-strand RNA virus of the recently reclassified *Pneumo­viridae* family (Afonso *et al.*, 2016 ▸) and is a major cause of lower respiratory tract infections in children, the elderly and the immunocompromised, including acute bronchiolitis and pneumonia (Reed *et al.*, 1997 ▸; Lay *et al.*, 2013 ▸; Amarasinghe *et al.*, 2017 ▸). HRSV is an enveloped virus; its genome consists of a 15.2 kb single-stranded nonsegmented negative-sense RNA that contains ten genes which encode 11 proteins. Four of them are part of the nucleocapsid: the nucleocapsid protein N, the polymerase cofactor phosphoprotein P, the transcription antitermination factor M_2–1_ and the viral polymerase L. The virus also encodes two non­structural proteins (NS1 and NS2) and three transmembrane proteins: the fusion F protein, the small hydrophobic SH protein and the G glycoprotein.

**Figure 1:**
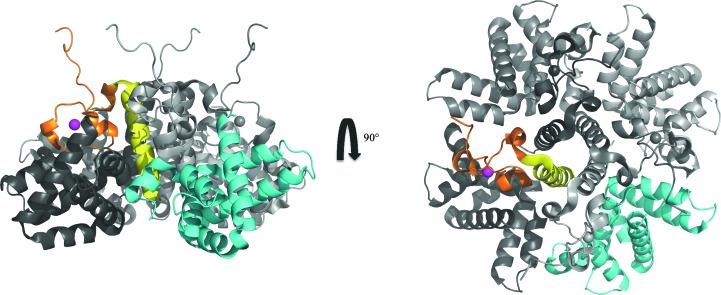
Domain interactions in tetrameric full-length HRSV M_2–1_. One monomer within the tetrameric full-length protein is coloured; the other three monomers are displayed in grey tones. The tetramerization domain is coloured yellow, the zinc-binding domain is in orange, the Zn^2+^ ion is in magenta and the RBD core domain is in cyan (PDB entry 4c3b).

The viral polymerase complex is responsible for carrying out genome replication, transcription and post-transcriptional modifications of mRNAs and consists of genomic RNA tightly bound to the nucleocapsid N protein and the P, L and M_2–1_ proteins (Collins *et al.*, 2001 ▸). The M_2–1_ transcription antiterminator factor is a basic protein of 194 amino acids that exists as a stable tetramer in solution (Tran *et al.*, 2009 ▸; Tanner *et al.*, 2014 ▸; Esperante *et al.*, 2011 ▸) and is essential for the complete sequential transcription of mRNAs in HRSV (Collins *et al.*, 1995 ▸, 1996 ▸; Yu *et al.*, 1995 ▸; Hardy & Wertz, 1998 ▸). It harbours four distinct regions: an N-terminal zinc-binding domain (ZBD; residues 7–25), an α-helical tetramer­ization domain (TD; residues 32–49), an RNA-binding core domain (RBD; residues 69–172) and a C-terminal unstructured region (residues 173–194). A crystal structure of the tetrameric M_2–1_ assembly (Tanner *et al.*, 2014 ▸) and an NMR structure of the monomeric M_2–1_ RBD (residues 58–177; Blondot *et al.*, 2012 ▸) have been determined. M_2–1_ oligomerization is primarily driven by a central tetramerization α-helix and is also stabilized by contacts between the zinc-binding domain and the core of an adjacent monomer (Tanner *et al.*, 2014 ▸; Fig. 1 ▸). The removal of zinc leads to tetramer dissociation into a monomeric apo M_2–1_ species in solution. Thus, the integrity of the ZBD seems to be critical for the tetrameric assembly and for protein function (Esperante *et al.*, 2013 ▸).

The crystallographic structure of the closely related *Human metapneumovirus* (HMPV) M_2–1_ protein revealed that the ZBD interacts with the tetramerization helix through a hydrophobic interface. The core domain interacts with the ZBD, and the tetramerization and core domains of adjacent protomers, mostly through polar contacts (Leyrat *et al.*, 2014 ▸). Interestingly, each protomer exists in an equilibrium between an open and a closed conformation. RNA binding induces the closed state, while the addition of subdenaturing concentrations of guanidinium chloride (GdmCl) induces an open state, consistent with the polar nature of the interactions between the core domain and the rest of the molecule (Leyrat *et al.*, 2014 ▸).

Given the relevance and uniqueness of this viral transcription antiterminator, we set out to investigate its RNA-binding domain (residues 73–194) using X-ray crystallography and folding thermodynamics. The structure of the monomeric domain is superimposable with that of the tetramer, but uncovers additional structured residues at the C-terminus that have not been observed previously. The folding studies reveal that although the RBD shows inter-monomer contacts and contacts the ZBD from neighbouring monomers in the tetramer, these do not influence its structure or stability.

## 2. Materials and methods   

### 2.1. Protein expression and purification   

The HRSV M_2–1_ protein from strain A was recombinantly expressed in bacteria and purified as described previously (Esperante *et al.*, 2013 ▸). The highly purified full-length M_2–1_ tetramer was then subjected to limited proteolysis using chymotrypsin from bovine pancreas (Sigma–Aldrich) at a 1:60(*w*:*w*) protein:chymotrypsin ratio in 50 m*M* Tris–HCl pH 8.0 for 90 min at 28°C. The reaction was stopped by adding 1 m*M* phenylmethylsulfonyl fluoride (PMSF) and subsequently subjected to size-exclusion chromatography on a Superdex 75 column in 20 m*M* Tris–HCl pH 7.0, 300 m*M* NaCl. The resulting RBD protein after proteolysis comprises amino acids Ala73–Tyr194. Its molecular weight of 13.6 kDa was confirmed by MALDI–TOF mass spectrometry (Bruker, Daltonics, Billerica, Massachusetts, USA) and the protein elutes as a monomeric peak from size-exclusion chromatography (Supplementary Fig. S2). The protein concentration was determined spectrophotometrically using an extinction coefficient (∊_280_) of 4470 *M* ^−1^ cm^−1^.

### 2.2. Folding and stability   

Equilibrium denaturation experiments were performed by incubating a 5 µ*M* protein sample with increasing concentrations of guanidinium chloride (GdmCl) in 20 m*M* Tris–HCl pH 7.0. The samples were incubated for 16 h at 20°C prior to measurements. CD spectra were recorded on a Jasco 815 spectropolarimeter. The CD data were fitted to a two-state model as described previously (Pretel *et al.*, 2013 ▸). Fluorescence spectroscopy was carried out using a Horiba Fluoro­max-4 spectrofluorometer with excitation at 275 nm. Intensity at 310 nm, corresponding to tyrosine fluorescence, was plotted as a function of the denaturant concentration. All measurements were performed at 20°C.

### 2.3. Crystallization and data collection   

Initial M_2–1_ RBD crystallization conditions were identified using the vapour-diffusion technique in sitting drops in a high-throughput crystallization screen. The most promising hits were then optimized by mixing 1 µl M_2–1_ RBD solution (11 mg ml^−1^) with 1 µl reservoir solution and equilibrating against reservoir solution consisting of either condition 1 (2.1 *M* dl-malic acid pH 7) or condition 2 (2.4 *M* sodium malonate pH 7) (both from the JCSG *plus* screen) at 293 K. The crystals belonged to space groups *P*2 and *P*3_2_21, respectively, and both diffracted to better than 2 Å resolution. Crystals appeared after 1 d and grew to maximum dimensions of 0.6 × 0.8 × 1.0 mm within one week (Supplementary Fig. S1). Crystals were mounted and flash-cooled in liquid nitrogen with cryoprotection (by the addition of 20% glycerol to the crystallization solution). Diffraction data were collected to a resolution limit of 1.8–2.0 Å. Full data sets with an interval of 0.1° were collected for both crystal forms on the P14 beamline at EMBL/DESY, Hamburg, Germany. All data sets were processed with *XDS* (Kabsch, 2010 ▸) and merged with *AIMLESS* (Evans, 2006 ▸). A summary of the data statistics is given in Table 1 ▸.

### 2.4. Structure determination, refinement and analysis   

The structures were solved by molecular replacement with *Phaser* (McCoy *et al.*, 2007 ▸) using PDB entry 4c3b (Tanner *et al.*, 2014 ▸) as the search model. Subsequent rounds of manual building using *Coot* (Emsley & Cowtan, 2004 ▸) and refinement using *phenix.refine* (Afonine *et al.*, 2012 ▸) allowed the model building of a short C-terminal extension of the M_2–1_ RBD (residues 73–194) in space group *P*3_2_21. The final models yielded crystallographic *R* factors of 17 and 19% and free *R* factors of 21 and 23% for the crystals in space groups *P*2 and *P*3_2_21, respectively (see Table 1 ▸ for details). The models were validated using *MolProbity* (Chen *et al.*, 2010 ▸). Evaluation of the Ramachandran plot showed all residues to be in allowed regions (97–99% in favoured regions). All figures were prepared using *PyMOL* (DeLano, 2002 ▸). The data have been deposited in the Protein Data Bank with PDB codes 5noh (*P*2) and 5nkx (*P*3_2_21).

### 2.5. Small-angle X-ray scattering (SAXS)   

SAXS data were collected on the P12 beamline at EMBL/DESY, Hamburg following standard procedures. Repetitive data collection from the same sample was performed and no radiation damage was detected. Samples of M_2–1_ RBD (residues Ala73–Tyr194) solution were prepared in the concentration range 4.5–9.0 mg ml^−1^ in a buffer consisting of 20 m*M* Tris–HCl pH 7, 300 m*M* NaCl. All SAXS data were analyzed using the *ATSAS* package (Franke *et al.*, 2017 ▸). Raw data were processed using *PRIMUS* (Konarev *et al.*, 2003 ▸). The radius of gyration (*R*_g_) was evaluated using the Guinier approximation [*I*(*s*) = *I*(0)exp(−*s* ^2^ *R*_g_^2^/3) for *sR*_g_ < 1.3] and also from the entire scattering curve with *GNOM* (Svergun, 1992 ▸); the latter also provided the distance distribution function *p*(*r*) and the maximum dimension *D*_max_. The masses of the solutes were evaluated by comparison of the forward scattering intensity with that from a BSA reference solution (66 kDa). Low-resolution SAXS models were obtained using the *ab initio* simulated-annealing (SA) program *DAMMIF* (Franke & Svergun, 2009 ▸), which generates models consisting of dummy atoms to fit the experimental data.

## 3. Results and discussion   

**Figure 2:**
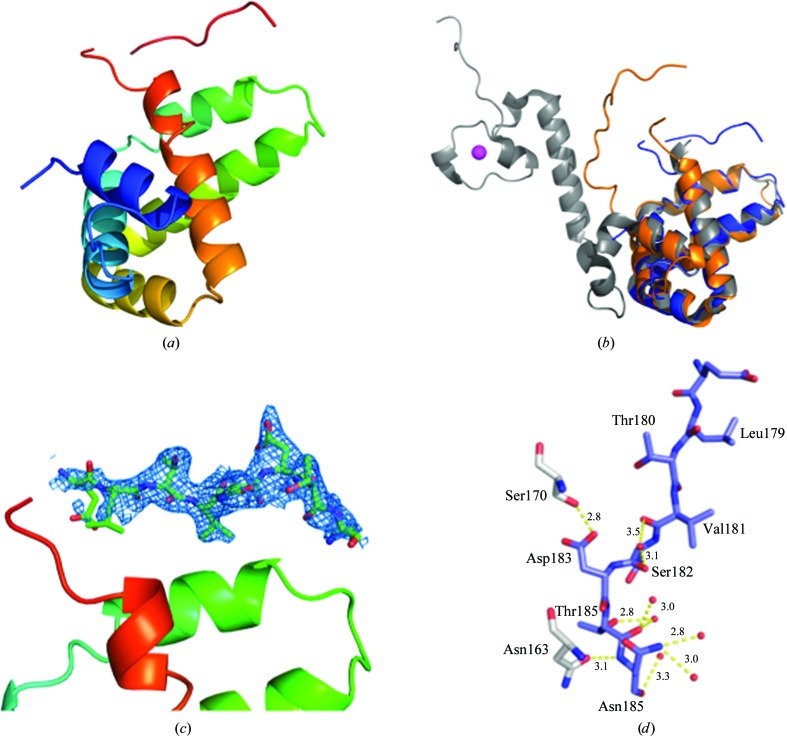
Overall structure of the HRSV M_2–1_ RBD. (*a*) Cartoon representation of the HRSV M_2–1_ RBD rainbow-coloured from the N-terminus (blue) to the C-­terminus (red). (*b*) Superposition of the HRSV M_2–1_ RBD crystal structure (blue) with the NMR structure (orange) and the crystal structure of the corresponding domain in the tetrameric assembly (grey). (*c*) Composite OMIT map of the C-terminal tail (residues 178–185) in the HRSV M_2–1_ RBD structure (*P*3_2_21 crystal form) contoured at 1.5σ. The colour code and orientation are as in (*a*), except for the C-terminal tail, which is displayed according to atom type. (*d*) Close-up of the C-terminal tail with contacting residues, waters and bond lengths.

The HRSV M_2–1_ RBD domain crystallized in two different space groups (Table 1 ▸) and the structure could be determined to 2.0 Å resolution. The structure consists of six α-helices connected by short loops (Fig. 2 ▸ *a*). The structures from both crystal forms are virtually identical in the core region (residues 58–177), with a root-mean-square deviation (r.m.s.d. on C^α^ atoms) of 0.3 Å. They are also very similar to the core domains observed in the crystal structure of full-length tetrameric HRSV M_2–1_ (Tanner *et al.*, 2014 ▸; r.m.s.d. of 0.7 Å) and in the NMR structure (Blondot *et al.*, 2012 ▸; r.m.s.d. of 1.39 Å) (Fig. 2 ▸ *b*). A notable exception is the C-terminal region (residues 178–185), which could be clearly modelled in one monomer in the *P*3_2_21 crystal form. In the tetrameric HRSV M_2–1_ crystal structure this region was disordered and thus not visible (Tanner *et al.*, 2014 ▸). In our *P*3_2_21 core-domain structure it is located in a cleft between helices H3 and H6 and occupies a position that would interfere with the zinc-binding domain (ZBD) of another monomer in the tetrameric assembly (Figs. 2 ▸ *c* and 3 ▸). We have shown that removing the zinc leads to a monomeric ‘apo’ form, which otherwise contains the same secondary structure as the tetrameric species, indicating that the ZBD modulates M_2–1_ oligomerization. In fact, this tetramer–monomer transition is increased at pH 5.5, strongly suggesting that the ZBD acts as a pH sensor that modulates quaternary structure (Esperante *et al.*, 2013 ▸).

**Figure 4:**
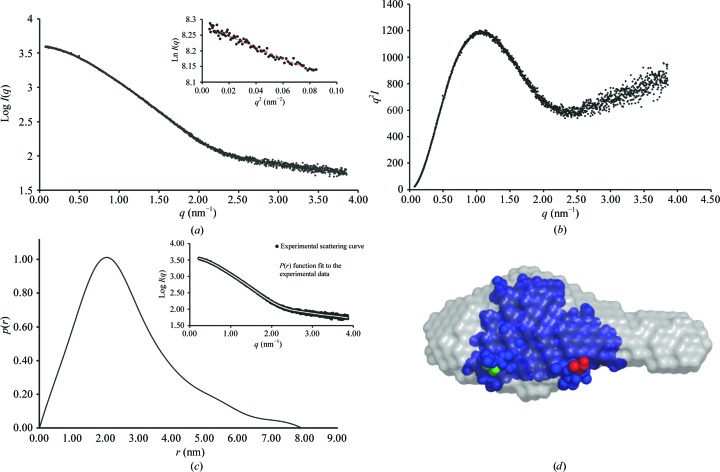
Small-angle X-ray scattering (SAXS) analysis of the M_2–1_ RBD. (*a*) Raw scattering profile of the HRSV M_2–1_ RBD with the Guinier region shown in the inset. (*b*) Kratky plot suggesting the presence of a folded core with a flexible tail. (*c*) Distance distribution plot. (*d*) *Ab initio* shape reconstruction (grey) superimposed on the crystal structure of the HRSV M_2–1_ RBD (PDB entry 5noh; blue). The N- and C-termini of the protein are highlighted in green and red, respectively.

The HRSV M_2–1_ RBD crystallized with two molecules in the asymmetric unit. The elution profile from size-exclusion chromatography indicates that the HRSV M_2–1_ RBD exists as a monomer in solution (Supplementary Fig. S2). However, in order to confirm the oligomeric state of the HRSV M_2–1_ RBD in solution at the high concentration used in the crystallization conditions, we performed small-angle X-ray scattering (SAXS) experiments. Model-free analysis of the SAXS data yielded an *R*_g_ of 20.3 Å from the Guinier plot (Fig. 4 ▸ *a*) and a Porod volume of 26.4 nm^3^, indicating a molecular weight of 15 kDa. The pair distance distribution function indicates a globular particle with a tail, with maximum dimensions (*D*_max_) of 8 nm (Fig. 4 ▸ *c*). The *ab initio* model of RBD obtained using *DAMMIF* is comparable to that of the crystal structure monomer (Fig. 4 ▸ *d*). The C-terminal tail observed in our *P*3_2_21 model could be extended in solution and would therefore explain the lack of globularity in our SAXS model. Electron density for this region has not been observed in previous crystal structures.

**Figure 5:**
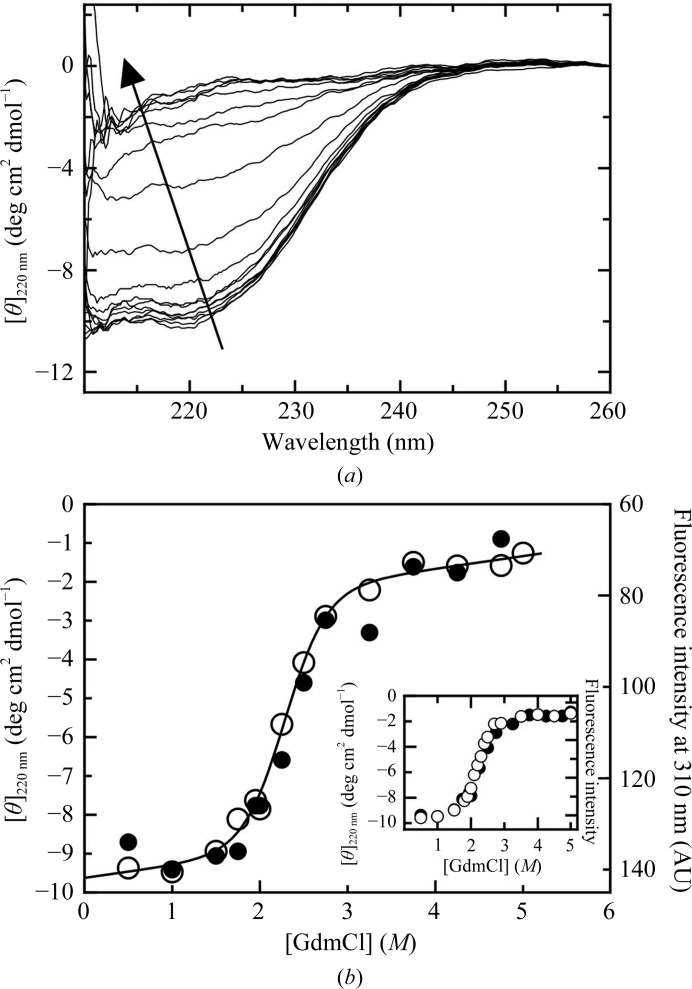
Unfolding transitions of the M_2–1_ RBD. (*a*) Far-UV CD spectra of the M_2–1_ RBD at increasing concentrations of GdmCl from 0 to 5 *M*, as indicated by the arrow. (*b*) RBD equilibrium unfolding transition followed by the molar ellipticity at 220 nm (empty circles) and tyrosine fluorescence at 310 nm (filled circles). Ellipticity data were fitted to a two-state unfolding model (see §[Sec sec2]2). Inset: comparison of GdmCl equilibrium unfolding of apo M_2–1_ (empty circles; Esperante *et al.*, 2011 ▸) with the M_2–1_ RBD (filled circles) monitored by the changes in molar ellipticity at 220 nm. Experiments were carried out at pH 7.0 (see §[Sec sec2]2).

The RNA-binding core domain appears to be a compact and cooperative folding unit in the crystal structure reported here and the previous NMR structure of a similar fragment (Tanner *et al.*, 2014 ▸). However, there are several intermonomer contacts in the tetramer, and other contacts connecting with other regions of the protein, which raises the questions of whether the domain is stabilized by such inter­actions and to what extent the domain is compact and stable in solution. We carried out equilibrium unfolding experiments using guanidine chloride, and followed the secondary-structure transition by monitoring changes in circular dichroism (CD) in the far-UV region. The mostly α-helical spectrum with the characteristic minima at 208 and 222 nm is gradually lost upon an increase in denaturant, indicating complete unfolding of the protein (M_2–1_ RBD; Fig. 5 ▸ *a*). The molar ellipticity change at 222 nm shows a highly cooperative transition suggestive of a two-state transition (Fig. 5 ▸ *b*). Since there are no tryptophan residues, we used tyrosine intrinsic fluorescence as a probe for tertiary structure, which produces a transition superimposable with the secondary structure, which is a strong indication of a two-state unfolding process (Fig. 5 ▸ *b*). For a quantitative analysis of the thermodynamic stability, we fitted the ellipticity data to a two-state model (Pretel *et al.*, 2013 ▸) and obtained an unfolding free-energy change (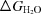) of 5.21 ± 0.92 kcal mol^−1^ and an *m* value of 2.28 ± 0.38 kcal mol^−1^ *M* ^−1^; the latter matches that expected for a globular two-state folder of this size (Myers *et al.*, 1995 ▸).

We have previously shown that zinc removal dissociates the tetramer, and the ‘apo’ M_2–1_ monomer obtained showed a secondary structure surprisingly identical to that of the tetramer (Esperante *et al.*, 2013 ▸). The unfolding transition of the M_2–1_ RBD is superimposable on that of the apo M_2–1_ monomer (Fig. 5 ▸ *b*, inset), with a 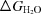 of 5.89 ± 0.33 kcal mol^−1^ and an *m* value of 2.62 ± 0.15 kcal mol^−1^ *M* ^−1^, which is in excellent agreement with the stability and *m* value of the monomeric RBD, confirming that the isolated monomeric RNA-binding domain is a folding unit that is independent of the rest of the molecule either as a tetramer or an apo monomer. These results indicate that the rest of the polypeptide (residues 1–72) is disordered when the zinc is removed and the tetramer dissociates, or at least there is no cooperative compact structure.

The fact that the M_2–1_ RBD is a stable and compact domain showing two-state cooperative unfolding, with identical thermo­dynamic stability to the apo M_2–1_ monomer, shows that the region that is missing in the isolated domain (residues 1–72) does not partake in any persistent structure, is not connected to the M_2–1_ RBD and forms the tetrameric interface only when the zinc-binding motif is intact (Esperante *et al.*, 2013 ▸; Fig. 1 ▸). Although only one highly symmetric structure was observed in the crystal structure of the HRSV M_2–1_ tetramer (Tanner *et al.*, 2014 ▸), the homologue from *Human metapneumovirus* (HMPV) showed open and closed conformations in which different RBDs move away from the tetrameric arrangement, and this conformational change was proposed to be the result of RNA binding (Leyrat *et al.*, 2014 ▸). The structure of the HMPV RBD does not change in the open or closed conformations, suggesting that there must be a flexible regulatory region which moves the RBD back and forth. In this picture, contacts of the RBD with other RBDs or other regions (*i.e.* the zinc-binding motif) must be regulatory, with no effect on RBD conformation or stability. In the HRSV tetramer, three of the chains show a lack of electron density between the tetramerization domain and the RBD (Tanner *et al.*, 2014 ▸), coincident with what could constitute a flexible hinge region that may modulate the open–closed transition. In the HMPV tetramer structure the same region also lacks electron density in the linker region between the TD and RBD, indicating high flexibility (as reflected by very high *B* factors for adjacent residues). On the other hand, the crystal structures presented here strongly suggest that the carboxy-terminal region of the protein would be unstructured in the closed conformation present in the RNA-free tetramer and folds upon the helical cleft corresponding to the ZBD-binding region in the RBD. In any case, the structures of both complexes with RNA plus mechanistic studies of the interaction with RNA are required for a complete picture of this puzzling transcription antiterminator activity that governs the relative viral protein levels, as determined by the action of the M_2–1_ tetramer unique to these two viruses within the pneumo­virus family.<!?tpb=-6pt>

## Supplementary Material

PDB reference: HRSV M_2–1_ core, 5nkx

PDB reference: 5noh

Supplementary Figures S1 and S2.. DOI: 10.1107/S2053230X17017381/ow5002sup1.pdf

## Figures and Tables

**Figure 3:**
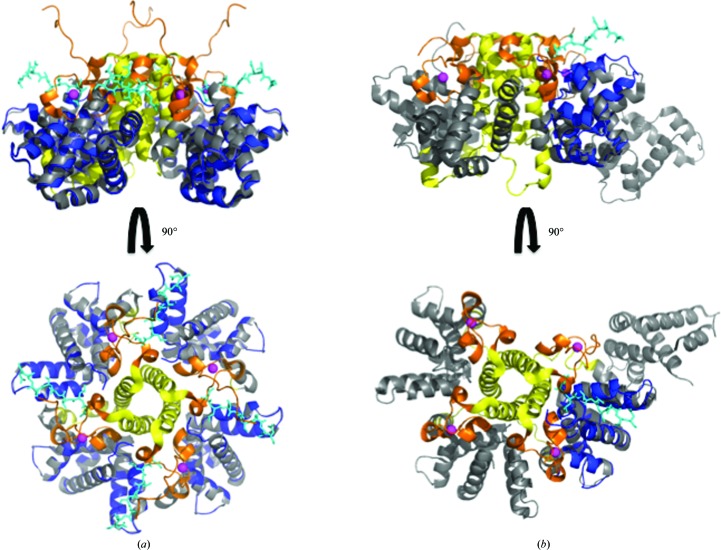
Structural comparison with tetrameric full-length HRSV M_2–1_ and HMPV M_2–1_. (*a*) Superposition of the HRSV M_2–1_ RBD (blue) with full-length HRSV M_2–1_. (*b*) Superposition of the HRSV M_2–1_ RBD (blue) with full-length HMPV M_2–1_. Within the full-length proteins, the tetramerization domains are coloured yellow, zinc-binding domains orange and the RBD core domains grey. Zn^2+^ ions are displayed as magenta spheres. The C-terminal tail (residues 178–185) in the HRSV M_2–1_ RBD structure that is not present in the tetrameric crystal structure is displayed as sticks and coloured cyan.

**Table 1:** Data-collection and refinement statistics Values in parentheses are for the highest resolution shell.

	HRSV M_2–1_ core (PDB entry 5noh)	HRSV M_2–1_ core (PDB entry 5nkx)
Data collection		
Space group	*P*2	*P*3_2_21
*a*, *b*, *c* (Å)	37.95, 69.24, 46.60	91.95, 91.95, 97.80
α, β, γ (°)	90, 90.05, 90	90, 90, 120
Resolution (Å)	46.60–1.80 (1.86–1.80)	41.67–2.00 (2.07–2.00)
*R*_merge_	0.050 (0.518)	0.043 (0.570)
〈*I*/σ(*I*)〉	12.1 (1.70)	10.0 (1.29)
CC_1/2_	0.99 (0.66)	0.99 (0.29)
Completeness (%)	94.8 (69.4)	99.98 (100)
Multiplicity	3.1 (2.4)	2.0 (2.0)
Refinement		
Resolution (Å)	1.8	2.0
No. of reflections	21206 (1591)	32735 (3196)
*R*_work_/*R*_free_	0.171/0.210	0.189/0.228
No. of atoms		
Total	1794	1857
Protein	1618	1668
Water	176	189
*B* factors (Å^2^)		
Protein	37.9	42.1
Water	45.3	50.4
R.m.s. deviations		
Bond lengths (Å)	0.007	0.003
Bond angles (°)	0.83	0.54

## References

[bb1] Afonine, P. V., Grosse-Kunstleve, R. W., Echols, N., Headd, J. J., Moriarty, N. W., Mustyakimov, M., Terwilliger, T. C., Urzhumtsev, A., Zwart, P. H. & Adams, P. D. (2012). *Acta Cryst.* D**68**, 352–367. 10.1107/S0907444912001308 PMC3322595 22505256

[bb2] Afonso, C. L. *et al.* (2016). *Arch. Virol.* **161**, 2351–2360. 10.1007/s00705-016-2880-1 PMC4947412 27216929

[bb3] Amarasinghe, G. K. *et al.* (2017). *Arch Virol.* **162**, 2493–2504. 10.1007/s00705-017-3311-7 PMC5831667 28389807

[bb4] Blondot, M.-L., Dubosclard, V., Fix, J., Lassoued, S., Aumont-Nicaise, M., Bontems, F., Eléouët, J.-F. & Sizun, C. (2012). *PLoS Pathog.* **8**, e1002734. 10.1371/journal.ppat.1002734 PMC3364950 22675274

[bb5] Chen, V. B., Arendall, W. B., Headd, J. J., Keedy, D. A., Immormino, R. M., Kapral, G. J., Murray, L. W., Richardson, J. S. & Richardson, D. C. (2010). *Acta Cryst.* D**66**, 12–21. 10.1107/S0907444909042073 PMC2803126 20057044

[bb6] Collins, P. L., Chanock, R. M. & Murphy, B. R. (2001). *Fields Virology*, 4th ed., edited by D. M. Knipe & P. M. Howley, pp. 1443–1486. Philadelphia: Lippincott, Williams & Wilkins.

[bb7] Collins, P. L., Hill, M. G., Camargo, E., Grosfeld, H., Chanock, R. M. & Murphy, B. R. (1995). *Proc. Natl Acad. Sci. USA*, **92**, 11563–11567. 10.1073/pnas.92.25.11563 PMC40442 8524804

[bb8] Collins, P. L., Hill, M. G., Cristina, J. & Grosfeld, H. (1996). *Proc. Natl Acad. Sci. USA*, **93**, 81–85. 10.1073/pnas.93.1.81 PMC40182 8552680

[bb9] DeLano, W. L. (2002). *PyMOL*. http://www.pymol.org.

[bb10] Emsley, P. & Cowtan, K. (2004). *Acta Cryst.* D**60**, 2126–2132. 10.1107/S0907444904019158 15572765

[bb11] Esperante, S. A., Chemes, L. B., Sánchez, I. E. & de Prat-Gay, G. (2011). *Biochemistry*, **50**, 8529–8539. 10.1021/bi200661k 21877705

[bb12] Esperante, S. A., Noval, M. G., Altieri, T. A., de Oliveira, G. A., Silva, J. L. & de Prat-Gay, G. (2013). *Biochemistry*, **52**, 6779–6789. 10.1021/bi401029q 23984912

[bb13] Evans, P. (2006). *Acta Cryst.* D**62**, 72–82. 10.1107/S0907444905036693 16369096

[bb22] Franke, D., Petoukhov, M. V., Konarev, P. V., Panjkovich, A., Tuukkanen, A., Mertens, H. D. T., Kikhney, A. G., Hajizadeh, N. R., Franklin, J. M., Jeffries, C. M. & Svergun, D. I. (2017). *J. Appl. Cryst.* **50**, 1212–1225. 10.1107/S1600576717007786 PMC5541357 28808438

[bb14] Franke, D. & Svergun, D. I. (2009). *J. Appl. Cryst.* **42**, 342–346. 10.1107/S0021889809000338 PMC5023043 27630371

[bb15] Hardy, R. W. & Wertz, G. W. (1998). *J. Virol.* **72**, 520–526. 10.1128/jvi.72.1.520-526.1998 PMC109403 9420254

[bb16] Kabsch, W. (2010). *Acta Cryst.* D**66**, 125–132. 10.1107/S0907444909047337 PMC2815665 20124692

[bb17] Konarev, P. V., Volkov, V. V., Sokolova, A. V., Koch, M. H. J. & Svergun, D. I. (2003). *J. Appl. Cryst.* **36**, 1277–1282.

[bb18] Lay, M. K., González, P. A., León, M. A., Céspedes, P. F., Bueno, S. M., Riedel, C. A. & Kalergis, A. M. (2013). *Microbes Infect.* **15**, 230–242. 10.1016/j.micinf.2012.11.012 23246463

[bb19] Leyrat, C., Renner, M., Harlos, K., Huiskonen, J. T. & Grimes, J. M. (2014). *Elife*, **3**, e02674. 10.7554/eLife.02674 PMC4051120 24842877

[bb20] McCoy, A. J., Grosse-Kunstleve, R. W., Adams, P. D., Winn, M. D., Storoni, L. C. & Read, R. J. (2007). *J. Appl. Cryst.* **40**, 658–674. 10.1107/S0021889807021206 PMC2483472 19461840

[bb21] Myers, J. K., Pace, C. N. & Scholtz, J. M. (1995). *Protein Sci.* **4**, 2138–2148. 10.1002/pro.5560041020 PMC2142997 8535251

[bb23] Pretel, E., Camporeale, G. & de Prat-Gay, G. (2013). *PLoS One*, **8**, e74338. 10.1371/journal.pone.0074338 PMC3769240 24058549

[bb24] Reed, G., Jewett, P. H., Thompson, J., Tollefson, S. & Wright, P. F. (1997). *J. Infect. Dis.* **175**, 807–813. 10.1086/513975 9086134

[bb25] Svergun, D. I. (1992). *J. Appl. Cryst.* **25**, 495–503.

[bb26] Tanner, S. J., Ariza, A., Richard, C.-A., Kyle, H. F., Dods, R. L., Blondot, M.-L., Wu, W., Trincão, J., Trinh, C. H., Hiscox, J. A., Carroll, M. W., Silman, N. J., Eléouët, J.-F., Edwards, T. A. & Barr, J. N. (2014). *Proc. Natl Acad. Sci. USA*, **111**, 1580–1585. 10.1073/pnas.1317262111 PMC3910626 24434552

[bb27] Tran, T.-L., Castagné, N., Dubosclard, V., Noinville, S., Koch, E., Moudjou, M., Henry, C., Bernard, J., Yeo, R. P. & Eléouët, J.-F. (2009). *J. Virol.* **83**, 6363–6374. 10.1128/JVI.00335-09 PMC2698528 19386701

[bb28] Yu, Q., Hardy, R. W. & Wertz, G. W. (1995). *J. Virol.* **69**, 2412–<!?show [forcelb]>2419. 10.1128/jvi.69.4.2412-2419.1995 PMC188915 7884888

