# Supplementary material for: Structure and stability of the Human respiratory syncytial virus M2–1 RNA-binding core domain reveals a compact and cooperative folding unit
Source: Acta Crystallogr F Struct Biol Commun. 2017 Dec 15;74(Pt 1):23–30. doi: 10.1107/S2053230X17017381 (PMC5947689; doi:10.1107/S2053230X17017381)
Supplement: Supplementary file 1 [file f-74-00023-sup1.pdf]

Acta Cryst F

STRUCTURAL BIOLOGY COMMUNICATIONS

Volume 74 (2018)

Supporting information for article:

Structure and stability of the *Human respiratory syncytial virus* M_{2-1} RNA-binding core domain reveals a compact and cooperative folding unit

Ivana G. Molina, Inokentijs Josts, Yasser Almeida Hernandez, Sebastian Esperante, Mariano Salgueiro, Maria M. Garcia Alai, Gonzalo de Prat-Gay and Henning Tidow

Supplementary Figure S1

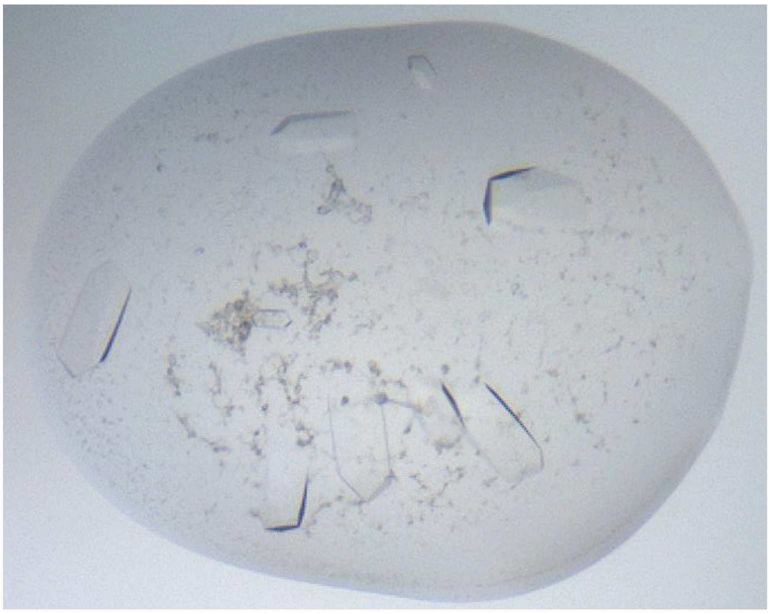

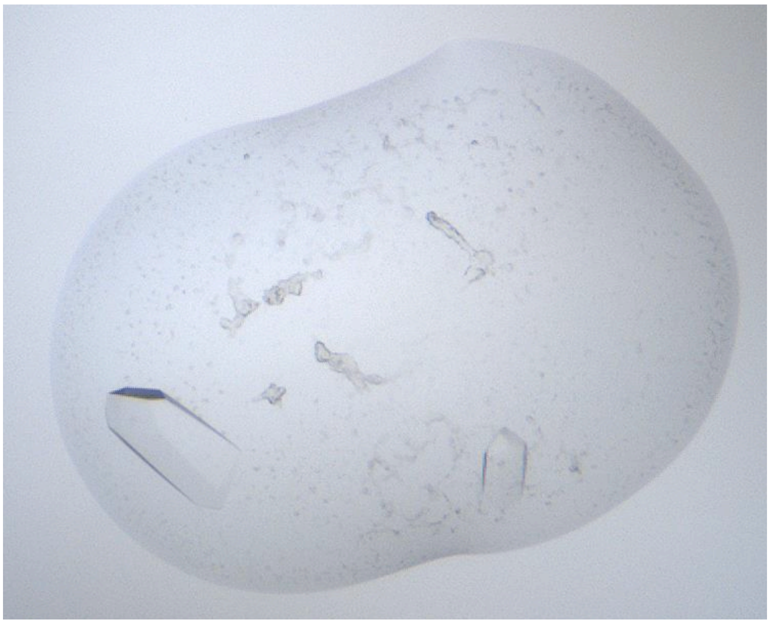

Representative crystals used for structure determination.

Supplementary Figure S2

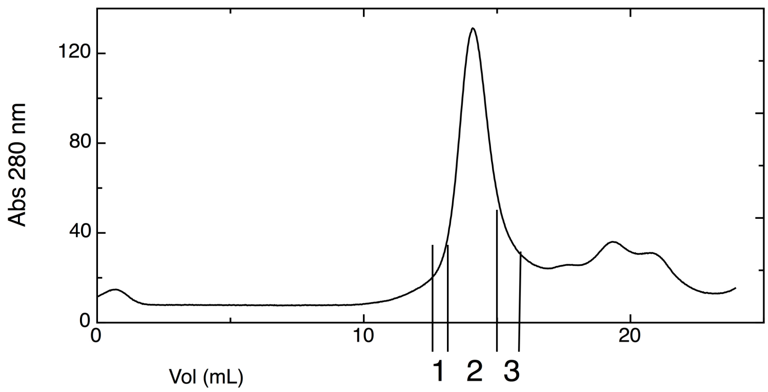

Size-exclusion chromatography (S75) profile of M_{2-1} RBD after proteolytic cleavage of full-length M_{2-1}. The molecular weight of the protein was 13.6 kDa.

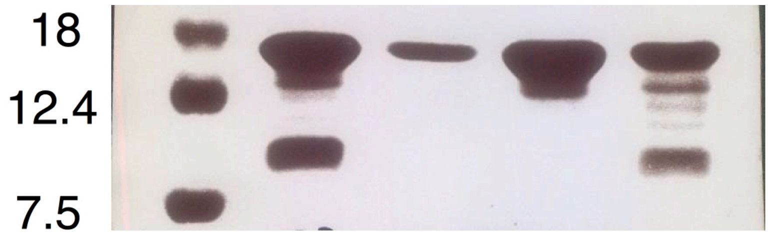

SDS-PAGE analysis of selected fractions of the size-exclusion chromatography.
